# Supplementary material for: A metagenomic DNA sequencing assay that is robust against environmental DNA contamination
Source: Nat Commun. 2022 Jul 21;13:4197. doi: 10.1038/s41467-022-31654-0 (PMC9304412; doi:10.1038/s41467-022-31654-0)
Supplement: Supplementary file 1 — Supplementary Information [file 41467_2022_31654_MOESM1_ESM.pdf]

Supplementary Information: A Metagenomic DNA Sequencing Assay that is Robust against Environmental DNA Contamination

Omary Mzava, Alexandre Pellan Cheng, Adrienne Chang, Sami Smalling, Liz-Audrey Kounatse Djomnang, Joan Sesing Lenz, Randy Longman, Amy Steadman, Luis G. Gomez-Escobar , Edward J. Schenck , Mirella Salvatore, Michael J. Satlin, Manikkam Suthanthiran, John R. Lee, Christopher E. Mason, Darshana Dadhania, Iwijn De Vlaminck

Supplementary Table 1. Overview of all the samples included in this study

**Cohort**	**Biofluid**		**Patients (n=150)**	**Samples (n=196)**
Kidney transplant	Urine		23	26
		UTI+		*16*
		UTI-		*10*
Early post-transplant	Urine		10	16
		Paired pre/post stent removal	5	10
Uganda	Plasma		44	56
		HIV+	9	11
		HIV-	35	45
COVID-19	Plasma		14	30
Sepsis	Plasma		15	15
		Sepsis	10	10
		Control	5	5
Inflammatory bowel disease	Plasma		44	53
		Non-IBD	4	4
		Ulcerative colitis	21	27
		Crohn's Disease	19	21
		Paired pre/post therapy	9	18
		Matched whole genome sequenced fecal samples	44	53

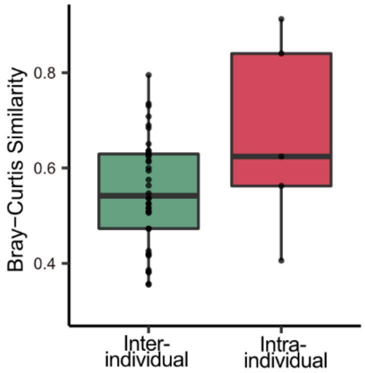

Supplementary Figure 1: Boxplot showing Bray-Curtis similarity index of the urine microbiome between patients and within individual patients before and after stent removal for the unfiltered datasets (Bray–Curtis Similarity 0.55± 0.11 and 0.67±0.2 respectively). Boxes in the boxplots indicates 25th and 75th percentile, the band in the box indicated the median and whiskers extend to 1.5 x Interquartile Range (IQR) of the hinge.

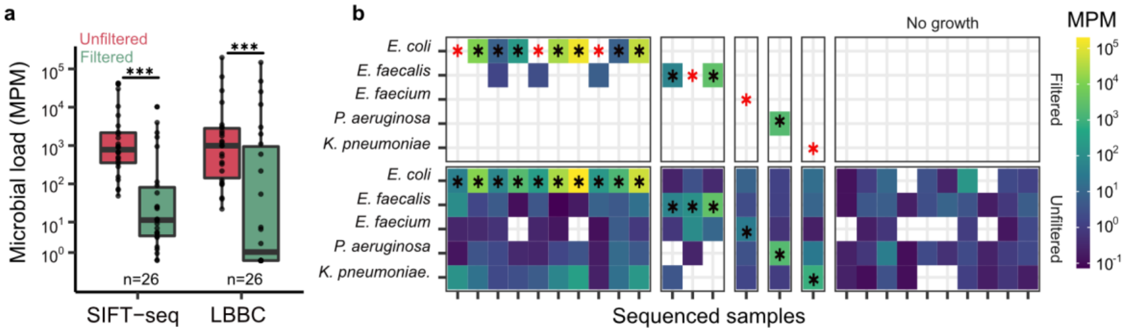

Supplementary Figure 2: Benchmarking SIFT-seq against Low Biomass Background Correction (LBBC). **A)** Boxplot of the total abundance (molecules per million, MPM) of contaminant genera before and after SIFT-seq or LBBC filtering (two tailed, Wilcoxon test, $P_{SIFT\text{-}seq} < 0.001$, $P_{\mathrm{LBBC}} < 0.001$) **B)** Heatmap of abundance of species (MPM) identified in patients with and without UTI, before and after application of LBBC filter. (Red * indicated species that were identified by culture and standard sequencing but removed after LBBC filtering). Boxes in the boxplots indicates 25th and 75th percentile, the band in the box indicated the median and whiskers extend to 1.5 x Interquartile Range (IQR) of the hinge. Outliers (beyond 1.5 × IQR) are plotted individually. *** p-value < 0.001

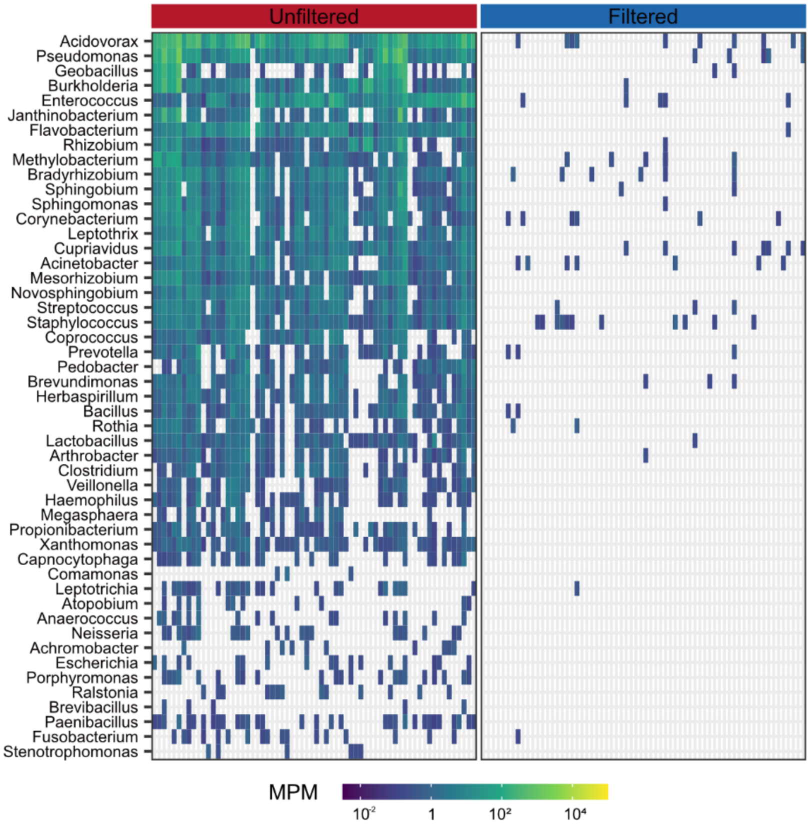

Supplementary Figure 3: Application of SIFT-seq filtering on negative, blank control samples

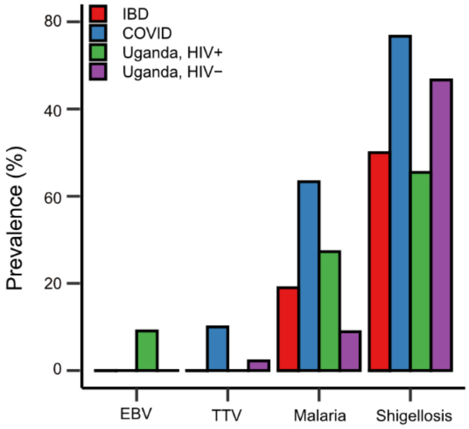

Supplementary Figure 4: Barplot of the prevalence of Epstein-Barr Virus (EBV), Torque teno virus (TTV), Malaria, or Shigellosis microorganisms in different patient cohorts before SIFT-seq filtering.

<u>Step-by-step protocol</u>

SIFT-seq in Urine

Reagents

- Lightning Conversion Reagent (Zymo Cat# D5032-1)
- L-Desulphonation Buffer (Zymo Cat# D5030-5)
- Urine Cell-Free Circulating DNA Purification Midi Kit (Norgen Cat #56700)
- UDI Primer Set-24 (SRSLY Cat# CBS-UD-24)
- PicoPlus DNA NGS Library Preparation Base Kit (SRSLY Cat# CBS-K250B-24)
- AmpureXP Beads (Beckman Coulter Cat# A63880)
- KAPA HiFi HotStart Uracil+ ReadyMix (2X) (Roche Cat# 7959052001)
- Qubit ssDNA Assay Kit (Invitrogen Cat# Q10212)

Equipment

- Two thermoshakers with 15 mL adapters
- Water bath
- Centrifuge

Step 1: Bisulfite Conversion

1. Preheat one thermoshaker to 98°C and set to 190 x g (1,000 RPM) (15s on, 30s off).
2. Preheat another thermoshaker to 54C and set to 190 x g (1,000 RPM) (15s on, 30s off).
3. Set the water bath to 55°C.
4. Thaw urine aliquots and spin down the samples for 5 minutes at 10°C and 20,000 x g (15,000 RPM).
5. Transfer 500ul of the supernatant to a 15 mL conical tube.
6. Add Lightning Conversion reagent to urine at a 6.5:1 ratio of reagent:supernatant.
7. Incubate in the 98°C thermoshaker for 10 minutes.
8. Incubate in the 54°C thermoshaker for 60 minutes.
9. Immediately proceed to next step

Step 2: cfDNA Extraction and Desulphonation

1. Complete the volume to 10 mL using 1x PBS.
2. Add 3 mL Binding Solution K and mix well by vortexing for 10 seconds.
3. Transfer 4.5 mL of the mixture to a MIDI Spin column assembled with one of the provided collection tubes.
4. Centrifuge for 3 minutes at 1,000 *x g*.
5. Discard the flowthrough and reassemble the spin column.
6. Repeat Steps 3-5 until all the fluid has passed.
7. Apply 3 mL of Wash Solution A to the column and centrifuge for 3 minutes at 1,000 *x g*. Discard flowthrough and repeat.
8. Apply 400 µL of Elution Buffer B to the column let stand at room temperature for 2 minutes. Centrifuge for 2 minutes at 500 *x g*.
9. Reapply the eluted 400 µL Elution Buffer B from Step 8 back to the column and let it stand at room temperature for 2 minutes. Centrifuge for 3 minutes at 500 *x g*.
10. Add 8 µL Proteinase K and mix well by vortexing for 10 seconds, then incubate at 55°C for 10 minutes.
11. After incubation, add 300 µL of Lysis Buffer A and mix well by vortexing for 10 seconds.

12. Add 400 µL of 96-100% ethanol, and mix well by vortexing for 10 seconds.
13. Transfer 750 µL of the mixture from Step 12 into a Mini Spin column assembled with one of the provided collection tubes. Centrifuge for 2 minutes at 3,300 *x g*.
14. Discard the flowthrough and reassemble the spin column with its collection tube.
15. Repeat Steps 13-14 one more time to transfer the remaining mixture into the Mini Spin column.
16. Apply 600 µL of Wash Solution A to the column and centrifuge for 1 minute at 3,300 *x g*. Discard the flowthrough and reassemble the spin column with its collection tube.
17. Repeat Step 16 one more time, for a total of two washes.
18. Add 200 uL of L-Desulphonation buffer and let it stand at room temperature for 15-20 minutes.
19. Centrifuge for 1 minute at 3,300 *x g*.
20. Add 600 uL of Wash solution A to the column and centrifuge for 1 minute at 3,300 *x g*.
21. Discard the flow-through.
22. Repeat Steps 20-21 for a total of two washes.
23. Spin the column empty for 2 minutes at 14,000 *x g* in a new collection tube.
24. Transfer the column to an elution tube.
25. Apply 30 uL of Elution Buffer B to the column and let stand at room temperature for 2 minutes.
26. Centrifuge for 1 minute at 200 *x g* for 1 minute, and then centrifuge for 2 minutes at 5,200 *x g*.
27. Transfer the eluate back to the column and let stand at room temperature for 2 minutes.
28. Centrifuge for 1 minute at 400 *x g*, followed by centrifugation for two minutes at 5,800 *x g*.
29. Quantify the abundance of the extracted single-stranded cfDNA.

SIFT-seq in Plasma

Reagents

- Lightning Conversion Reagent (Zymo Cat# D5032-1)
- L-Desulphonation Buffer (Zymo Cat# D5030-5)
- QIAamp Circulating Nucleic Acid Kit (Qiagen Cat# 55114)
- UDI Primer Set-24 (SRSLY Cat# CBS-UD-24)
- PicoPlus DNA NGS Library Preparation Base Kit (SRSLY Cat# CBS-K250B-24)
- AmpureXP Beads (Beckman Coulter Cat# A63880)
- KAPA HiFi HotStart Uracil+ ReadyMix (2X) (Roche Cat# 7959052001)
- Qubit ssDNA Assay Kit (Invitrogen Cat# Q10212)

Equipment

- Two thermoshakers with 15 mL adapters
- Water bath
- Centrifuge

Step 1: Bisulfite Conversion

1. Preheat one thermoshaker to 98°C and set to 1,000 RPM (15s on, 30s off).
2. Preheat another thermoshaker to 54°C and set to 1,000 RPM (15s on, 30s off).
3. Set the water bath to 60°C.

4. Thaw plasma aliquots and spin down for 5 mins at 20,000 x g (15,000 RPM) and 10°C.
5. Transfer 500 µL of the supernatant to a 15 mL conical tube.
6. Add 600 µL of 1X PBS.
7. Incubate at 98°C for 10 minutes with constant shaking at 190 x g (1,000 RPM).
8. Centrifuge at 5880xg(7,500 RPM) for 10 minutes.
9. Transfer supernatant to a new 15 mL tube.
10. Add Lightning Conversion reagent to plasma at a 6.5:1 ratio of reagent:supernatant.
11. Incubate in the 98°C thermoshaker for 10 minutes.
12. Incubate in the 54°C thermoshaker for 60 minutes.
13. Immediately proceed to next step

Step 2: cfDNA Extraction and Desulphonation

1. Add an appropriate amount of carrier RNA to Buffer ACL.
2. Add 400 µL of Proteinase K into a 50 mL centrifuge tube.
3. Complete the volume to 4 mL with 1X PBS and add to the 50 mL tube containing Proteinase K.
4. Add 3.2 mL Buffer ACL (with carrier RNA). Pulse vortex 30 seconds.
5. Incubate at 60°C for 30 minutes.
6. Place the tube back on the lab bench and unscrew the cap
7. Add 7.2 mL Buffer ACB to the lysate. Pulse vortex 15-30 seconds.
8. Incubate on ice for 5 minutes.
9. Add the mixture to the tube extender and turn on the vacuum.
10. Apply 600 µL ACW1. Drain the column.
11. Apply 750 µL ACW2. Drain the column.
12. Apply 750 µL 96-100% ethanol. Drain the column.
13. Add 200 µL of L-Desulphonation buffer. Close tops and incubate for 15-20 minutes. Drain the column/
14. Apply 750 µL 96-100% ethanol. Drain the column and repeat for a total of two washes.
15. Transfer the column to a collection tube and centrifuge at 20,000 x g (14,000) rpm for 3 minutes.
16. Transfer the column to a new collection tube and incubate on a heat block set at 56°C for 10 minutes with the lid open.
17. Place the column in a clean 1.5 mL elution tube. Add 25 µL of Buffer AVE to the center of the column. Close lid and incubate for 3 minutes at room temperature.
18. Centrifuge at 20,000 x g (14,000 rpm) for 1 minute.
19. Quantify the abundance of the extracted single-stranded cfDNA.[1]
