# Supplementary material for: A metagenomic DNA sequencing assay that is robust against environmental DNA contamination
Source: Nat Commun. 2022 Jul 21;13:4197. doi: 10.1038/s41467-022-31654-0 (PMC9304412; doi:10.1038/s41467-022-31654-0)
Supplement: Supplementary file 3 — Reporting Summary [file 41467_2022_31654_MOESM3_ESM.pdf]

nature portfolio

Corresponding author(s): Iwijn De vlaminck

Last updated by author(s): May 17, 2022

Reporting Summary

Nature Portfolio wishes to improve the reproducibility of the work that we publish. This form provides structure for consistency and transparency in reporting. For further information on Nature Portfolio policies, see our Editorial Policies and the Editorial Policy Checklist.

Statistics

For all statistical analyses, confirm that the following items are present in the figure legend, table legend, main text, or Methods section.

n/a	Confirmed	
[ ]	[x]	The exact sample size (*n*) for each experimental group/condition, given as a discrete number and unit of measurement
[ ]	[x]	A statement on whether measurements were taken from distinct samples or whether the same sample was measured repeatedly
[ ]	[x]	The statistical test(s) used AND whether they are one- or two-sided<br>*Only common tests should be described solely by name; describe more complex techniques in the Methods section.*
[ ]	[x]	A description of all covariates tested
[x]	[ ]	A description of any assumptions or corrections, such as tests of normality and adjustment for multiple comparisons
[ ]	[x]	A full description of the statistical parameters including central tendency (e.g. means) or other basic estimates (e.g. regression coefficient) AND variation (e.g. standard deviation) or associated estimates of uncertainty (e.g. confidence intervals)
[ ]	[x]	For null hypothesis testing, the test statistic (e.g. *F*, *t*, *r*) with confidence intervals, effect sizes, degrees of freedom and *P* value noted<br>*Give P values as exact values whenever suitable.*
[x]	[ ]	For Bayesian analysis, information on the choice of priors and Markov chain Monte Carlo settings
[x]	[ ]	For hierarchical and complex designs, identification of the appropriate level for tests and full reporting of outcomes
[ ]	[x]	Estimates of effect sizes (e.g. Cohen's *d*, Pearson's *r*), indicating how they were calculated

Our web collection on statistics for biologists contains articles on many of the points above.

Software and code

Policy information about availability of computer code

Data collection	We did not use any software for data collection
Data analysis	All data analysis scripts for this study has been made available on Github (https://github.com/omrmzv/SIFTseq). A detailed description of the pipeline has been provided in the supplementary methods section.<br><br>Sequence data processing and alignment:<br>Adapter and low quality bases from the reads were trimmed using BBDuk (BBDuk V38.46, --entropy= '0.25' --maq= '10' -Xmx1g tbo tpe) and aligned to the C-to-T and G-to-A converted human genome(hg19) using Bismark with default parameters (Bismark-0.22.1; --unmapped, --quiet). PCR duplicates were removed using Bismark (Bismark-0.22.1).<br><br>Depth of coverage:<br>The depth of sequencing was measured by summing the depth of coverage for each mapped base pair on the human genome after duplicate removal, and dividing by the total length of the human genome (hg19, without unknown bases).<br><br>Removing unconverted molecules:<br>Aligned BAM files are filtered to remove unconverted molecules using the Bismark(Bismark-0.22.1) alignment package with default parameters.<br><br>Bisulfite conversion efficiency:<br>We estimated bisulfite conversion efficiency by quantifying the rate of C[A/T/C] methylation in human-aligned reads (MethPipe3 V3.4.3; -m general -L 500), which are rarely methylated in mammalian genomes.

Pre-processing of the unmapped reads:
Reads originating from the Phix genome were removed from the host unmapped reads using Bowtie2 (Bowtie 2.4.3, --local, --very-sensitive-local, --un-conc). Read IDs from the remaining reads were used to subset paired end reads from the original FASTQ files. Adapter trimming and read quality filtering was performed using BBDuk1 (BBDuk V38.46, maq=32). Remaining reads were deduplicated using samtools5 and merged using FLASH2(-q -M75 -O). K-mer decontamination to remove human reads was then performed using BBDuk1 (k=50, prealloc = t) and the obtained fastq file was converted to a fasta file for metagenomics analysis.

Metagenomic abundance estimation from sequencing data:
Reads mapping to microbial species were identified using HS-BLASTN(hs-blastn-1.0.0) and microbial abundances estimated using GRAMMy (version 1). Read and species level filtering was performed using custom scripts written in Python.

Identification of translocated gut bacteria in plasma:
Fecal shotgun metagenomic data was trimmed using Trim Galore V0.6.5 (--nextera --paired) and aligned to the human genome using Bowtie 2 (Bowtie 2.4.3; --maxins 700 --no-discordant --score-min L,0,-0.2). Reads that did not align to the human genome were extracted and assembled using SPAdes (SPAdes 3.15.3; --meta). The assembled metagenomes were classified using Kaiju10 (Kaiju 1.7.4). Paired cfDNA reads obtained after SIFT-seq filtering were then aligned to the assembled metagenomes using Bismark (Bismark 0.22.1).

Benchmarking SIFT-seq against Low Background Biomass Correction(LBBC):
To benchmark SIFT-seq we compared its performance to Low Microbial Biomass Background Correction (LBBC) tool. Default LBBC filtering parameters were used for this analysis ($\Delta CVmax=2$, $\delta 2min=-5.5$). The standard sequencing data used for LBBC are available in the database of Genotypes and Phenotypes (dbGaP), accession number phs001564.v1.p1

Statistical analysis:
All statistical methods were performed in R version 4.0.5.

For manuscripts utilizing custom algorithms or software that are central to the research but not yet described in published literature, software must be made available to editors and reviewers. We strongly encourage code deposition in a community repository (e.g. GitHub). See the Nature Portfolio guidelines for submitting code & software for further information.

Data

Policy information about availability of data

All manuscripts must include a data availability statement. This statement should provide the following information, where applicable:

- Accession codes, unique identifiers, or web links for publicly available datasets
- A description of any restrictions on data availability
- For clinical datasets or third party data, please ensure that the statement adheres to our policy

ΦX174 DNA sequencing data used in the proof of principle experiments has been deposited in NCBI's Sequence Read Archive (SRA) under Bioproject ID PRJNA782310 [https://www.ncbi.nlm.nih.gov/sra?linkname=bioproject_sra_all&from_uid=782310]. Sequencing data from human plasma and urine cfDNA is available in the database of Genotypes and Phenotypes (dbGaP), accession number phs001564.v1.p1 [https://www.ncbi.nlm.nih.gov/projects/gap/cgi-bin/study.cgi?study_id=phs000645.v1.p1]

Field-specific reporting

Please select the one below that is the best fit for your research. If you are not sure, read the appropriate sections before making your selection.

☒ Life sciences ☐ Behavioural & social sciences ☐ Ecological, evolutionary & environmental sciences

For a reference copy of the document with all sections, see nature.com/documents/nr-reporting-summary-flat.pdf

Life sciences study design

All studies must disclose on these points even when the disclosure is negative.

Sample size	We collected as many patient samples as possible that fit the criteria in each cohort. A detailed description of the sample sizes in each cohort is given in supplementary Table 1.
Data exclusions	We excluded urine samples that had the following criteria.<br>1. We excluded data from 3 samples that were collected by Foley from the Kidney Transplant cohort samples, included samples were all collected by clean-catch method.<br>2. We excluded data from 4 samples that had mixed urine culture results or associated with positive urine culture from the Early Post Transplant cohort, included samples were all urine culture negative.
Replication	Given the cost of the omics assay developed we did not replicate individual measurements, but rather tested reproducibility by measuring many samples many cohorts of patients. For each of the two proof-of-principle experiments included in Figure 1, we measured six replicates with different biomass.

Randomization	Randomization is not relevant given that we developed an omics assay that measures many parameters simultaneously.
Blinding	The investigators were blinded to group allocation during data collection of samples in the Sepsis cohort. Groups, and detailed clinical information (e.g. data from conventional blood cultures) were shared with the investigators after the data was analyzed and shared with collaborators who then shared metadata elements. For the other groups, blinding was not implemented the study was focused on the development of a new method and because in the case of the Kidney transplant and Uganda cohorts group allocations were available from prior studies by the same investigators.

Reporting for specific materials, systems and methods

We require information from authors about some types of materials, experimental systems and methods used in many studies. Here, indicate whether each material, system or method listed is relevant to your study. If you are not sure if a list item applies to your research, read the appropriate section before selecting a response.

Materials & experimental systems

n/a	Involved in the study
☒	☐ Antibodies
☒	☐ Eukaryotic cell lines
☒	☐ Palaeontology and archaeology
☒	☐ Animals and other organisms
☐	☒ Human research participants
☒	☐ Clinical data
☒	☐ Dual use research of concern

Methods

n/a	Involved in the study
☒	☐ ChIP-seq
☒	☐ Flow cytometry
☒	☐ MRI-based neuroimaging

Human research participants

Policy information about studies involving human research participants

Population characteristics	Uganda cohort<br>44 plasma samples were collected from individuals seeking tuberculosis treatment in Uganda. This group consisted of 26 males and 18 females with ages between 18 and 54<br><br>IBD cohort:<br>Peripheral blood samples were collected from 21 patients with Ulcerative Colitis , 19 with Crohn's disease and 4 non-IBD patient. Among these 24 were females and 20 were males with ages between 19 and 67.<br><br>IBD cohort Stool sample collection:<br>Matched stools samples were collected for the patients with Inflammatory Bowel Disease.<br><br>COVID-19 cohort:<br>30 samples were collected from 14 patients as part of an observational study among individuals with COVID-19 that were treated at New York Presbyterian Hospital and Lower Manhattan Hospitals. This group consisted of 8 males and 6 females with ages between 33 and 85.<br><br>Sepsis cohort:<br>15 blood samples were collected from 15 patients in ICU (10 septic and 5 non septic, 7 males and 8 females with ages between 46 and 97)<br><br>Kidney Transplant Cohort:<br>26 urine samples were collected from 23 kidney(15 males and 8 females) transplant recipients who received care at New York Presbyterian Hospital–Weill Cornell Medical Center. This group consisted of patients with ages between between 26 and 83.<br><br>Early Post Transplant Cohort:<br>16 Urine samples were collected from 10 patients within 47 ± 11 days post-kidney transplantation. This group consisted of 7 males and 3 females with age between 36 and 72.<br><br>A detailed information of all the samples included in this study is given in supplementary Table 1.
Recruitment	This is a methods focused study with a retrospective study design. Samples were selected from a biobank to represent a range of disease states, e.g. samples matched with a corresponding positive or negative culture result.
Ethics oversight	Uganda Cohort.<br>This study was approved by the Makerere School of Medicine Research and Ethics Committee (protocol 2017-020)<br><br>IBD cohort.

Peripheral blood and stool samples were collected under IRB approved protocol (1806019340) at the Jill Roberts Center for IBD at Weill Cornell Medicine

COVID 19 cohort.
This study was approved by the Institutional Review Board of Weill Cornell Medicine (IRB 20-03021645), and informed consent was obtained from all participants.

Sepsis cohort
This was approved by the institutional review board of Weill Cornell Medicine (1405015116, 20-05022072)

Kidney Transplant Cohort:
This study was approved by the Weill Cornell Medicine Institutional Review Board (protocols 1207012730)

Early Post Transplant Cohort:
This study was approved by the Weill Cornell Medicine Institutional Review Board (protocol 20-01021269).

Note that full information on the approval of the study protocol must also be provided in the manuscript.
